# Supplementary material for: Waist rotation angle as indicator of probable human collision-avoidance direction for autonomous mobile robots
Source: PLoS One. 2025 May 14;20(5):e0323632. doi: 10.1371/journal.pone.0323632 (PMC12077734; doi:10.1371/journal.pone.0323632)
Supplement: S1 — (DOCX) [file pone.0323632.s001.docx]

**Supporting Information**

Waist rotation angle as indicator of probable human collision-avoidance direction for autonomous mobile robots

Tatsuto Yamauchi, Hideki Tamura, Tetsuto Minami, Shigeki Nakauchi

**Appendix**

**Experiment 1**

**Participants**

Sixteen students (one female and fifteen male) from Toyohashi University of Technology participated in this experiment, with an average age of 22.9 ± 0.7 years. This sample size aligns with that of a previous study [1]. All participants were right-handed, with an average hand preference score of 9.1 ± 1.9, as assessed by the Flinders Handedness Survey (FLANDERS) questionnaire [2,3]. All experimental protocols involving human participants were approved by the institutional review board of Toyohashi University of Technology (2021-2), in accordance with the Declaration of Helsinki. Written informed consent was obtained from all participants for the publication of their details. The experiment was conducted from November 10 to November 26, 2021.

**Apparatus**

**Control system for AMR:** The AMR dimensions were 460 mm × 320 mm × 800 mm (length × width × height). The onboard computer controlled two motors connected to the axles of the wheels. Another computer in the experimental chamber received signals from a tracking system (described below) and transmitted them to the onboard computer via Wi-Fi. The robot operating system (ROS) Melodic with Ubuntu 18.04 LTS controlled both computers. Participants were unfamiliar with this AMR, ensuring unbiased results. Notably, the AMR was not equipped with any obstacle-avoidance algorithms during Experiment 1; it functioned solely as a moving agent.

**Tracking system:** A computer tracking system running Windows 10 communicated with the trackers, managed via Unity (2020.3.20f1), Steam VR (1.20.4), and Steam VR Unity Plugin (v2.7.3; SDK 1.14.15). The system detected the position and rotation of each body part at a 90 Hz sampling frequency within a play area defined by four base stations (Steam VR Base Station 2.0) in the experimental room.

**Experiment 2**

**Participants**

Nineteen students from Toyohashi University of Technology (one female, eighteen male; average age 22.5 ± 1.3 years) participated in this experiment, matching the sample size of Experiment 1. All participants were right-handed, with an average hand preference score of 9.4 ± 1.5, as determined by the same questionnaire used in the previous experiment. The institutional review board of Toyohashi University of Technology approved the experimental protocols (approval number 2021-02), adhering to the Declaration of Helsinki. Written informed consent for the publication of details was obtained from each participant. The experiment took place from July 7, 2022, to July 21, 2022.

**Experiment 3**

**Participants**

Twenty students from Toyohashi University of Technology participated in the study (five female, fifteen male; average age 22.0 ± 0.76 years). However, one participant was excluded due to their height, which impeded the camera's ability to capture their body posture, leaving nineteen students for analysis. All participants were right-handed, with an average hand preference score of 9.5 ± 1.5, determined using the same questionnaire as in previous studies. The institutional review board of Toyohashi University of Technology approved all experimental protocols (2023-22) in accordance with the Declaration of Helsinki. Written informed consent for publication of details was obtained from each participant. The experiment was conducted from May 10, 2024, to May 22, 2024.

**Apparatus**

**Control system for AMR:** In Experiment 3, we replaced the AMR used in Experiments 1 and 2 with a new AMR compatible with ROS2. At the time of preparation, ROS1 was no longer supported, and ROS2, which relies on Python 3, was required for implementing pose estimation. The dimensions of the AMR were 330 mm × 270 mm × 800 mm (length × width × height). The onboard computer of the AMR controlled two motors connected to the axles of its wheels. Another computer in the experimental chamber received signals from a tracking system (described below) and transferred them to the onboard computer via Wi-Fi. The Robot Operating System 2 (ROS2) Humble, running on Ubuntu 22.04 LTS, was used to control both computers. The MediaPipe Pose model bundle used was Pose Landmarker (Full), with the configuration option min_pose_detection_coherence set to 0.8 and all other settings left as default.

**Tracking system:** The tracking system and motion tracker placement were the same as in Experiments 1 and 2. However, due to updates in ROS2, we used updated software versions. The computer tracking system (running Windows 11) communicated with the trackers via Unity (2022.3.21f1), Steam VR (2.5.5), and the Steam VR Unity Plugin (v2.8.0; SDK 2.0.10). The system detected the position and rotation of each body part at a 90 Hz sampling frequency within a play area defined by four base stations (Steam VR Base Station 2.0) in the experimental room.

**References**

1. Souza Silva W, Aravind G, Sangani S, Lamontagne A. Healthy young adults implement distinctive avoidance strategies while walking and circumventing virtual human vs. non-human obstacles in a virtual environment. Gait Posture. 2018;61: 294–300. doi: [10.1016/J.GAITPOST.2018.01.028](https://doi.org/10.1016/j.gaitpost.2018.01.028).
2. Okubo M, Suzuki H, Nicholls MER. A Japanese version of the FLANDERS handedness questionnaire. Jpn J Psychol. 2014;85: 474–481. doi: [10.4992/JJPSY.85.13235](https://doi.org/10.4992/jjpsy.85.13235).
3. Nicholls MER, Thomas NA, Loetscher T, Grimshaw GM. The Flinders Handedness survey (Flanders): A brief measure of skilled hand preference. Cortex. 2013;49: 2914–2926. doi: [10.1016/J.CORTEX.2013.02.002](https://doi.org/10.1016/j.cortex.2013.02.002).
