# Supplementary material for: Waist rotation angle as indicator of probable human collision-avoidance direction for autonomous mobile robots
Source: PLoS One. 2025 May 14;20(5):e0323632. doi: 10.1371/journal.pone.0323632 (PMC12077734; doi:10.1371/journal.pone.0323632)
Supplement: Attachment — Submitted filename: Yamauchi_mobility_response_R2_20240918.docx [file pone.0323632.s002.docx]

**Response Letter**

We thank the editor and reviewers for the time and effort dedicated to reviewing our manuscript. The changes we have implemented based on their insightful comments, suggestions, and feedback have significantly improved our work. We have made significant revisions to strengthen the manuscript, including the addition of a new Experiment 3, which further enhances the value of our findings. We have also thoroughly revised the structure of the manuscript to improve clarity and flow. Moreover, we have utilized a professional English language editing service to comprehensively improve the language quality of the manuscript. Detailed responses to the reviewers’ comments are provided below.

-----

Reviewer #1:

[Rev#1C1] Dear editors and authors,

A Waist rotation angle is studied as an indicator of probable human collision avoidance direction for autonomous mobile robots. This manuscript describes psychophysical experiments and in order to improve its quality I nicely request the authors to consider my comments:

[Rev#1R1] Thank you for summarizing our paper and for providing your suggestions for improvement.

[Rev#1C2] 1) Make your data available: Your Data (tracker_S01.mat, tracker_S02.mat etc.) uploaded at https://osf.io/ajymd/, but cannot be downloaded!

[Rev#1R2] Thank you for bringing this to our attention. We have confirmed that the data is available for download. Please follow these steps to access the files: select “OSF Storage (United States)” and click the “Download as zip” button (see the figure below).

[Rev#1C3] 2) Reorganize the structure of your paper: Section, Subsections.

[Rev#1R3] Thank you for your suggestion. Accordingly, we have implemented the following revisions:

1. **Introduction:** The structure has been revised for greater clarity, with a more explicit presentation of the background and objectives. Please also refer to Rev#1R4.
2. **Literature Review:** A new section has been added to provide a comprehensive review of relevant research. Please also refer to Rev#1R5.
3. **Discussion:** The individual discussions pertaining to each experiment have been consolidated into a single Discussion section, with subsections added for improved clarity.

These changes are intended to enhance the overall flow and readability of the paper.

[Rev#1C4] 3) Improve the introduction section and add at the end a paragraph to describe the reminder sections of the paper.

[Rev#1R4] Thank you for your recommendation. We have enhanced the Introduction section by incorporating new subsections (*Human robot interaction*, *Robot control using AI methods*, *Cognitive science towards robots*, *Avoidance behavior between humans and robots*, and *Aims of this study*) and adding a paragraph delineating the structure of the paper (p. 6: lines 115-125).

[Rev#1C5] 4) Add a literature review section (discard the literature review from the introduction).

[Rev#1R5] Thank you for your specific suggestion. We have added a literature review section following the introduction, relocating several relevant paragraphs from the previous version of the introduction to this new section (pp. 6-9: lines 127-180).

[Rev#1C6] 5) It is mandatory to add a new section to describe the artificial intelligence methods dedicated for the autonomous mobile robot.

[Rev#1R6] Thank you for your constructive feedback. We have added a new subsection titled “Robot control using AI methods” to the introduction section (p. 4: lines 63-76).

[Rev#1C7] 6) Enrich your bibliographies with the fastest method (entitled DM-SPP) for the autonomous mobile robot. More details of the rapid DM-SPP are available at:

6.1.) Dhouib S. (2023) “Shortest Path Planning via the Rapid Dhouib-Matrix-SPP (DM-SPP) Method for the Autonomous Mobile Robot”. Results in Control andOptimization, Vol. 13, ID: 100299, DOI: 10.1016/j.rico.2023.100299.

6.2.) Dhouib S. (2024) “Faster than Dijkstra and A* Methods for the Mobile Robot Path Planning Problem Using Four Movement Directions: The Dhouib-Matrix-SPP-4”.Advances in Transdisciplinary Engineering, Mechatronics and Automation Technology, Vol. 46, pp. 284-290, DOI: 10.3233/ATDE231116.

[Rev#1R7] Thank you for bringing these papers to our attention, which we have now cited in the Introduction section (p. 4: lines 70-72).

[Rev#1C8] 7) Improve the experimental study with other case studies.

[Rev#1R8] Thank you for your valuable feedback on enhancing the experimental study with additional case studies. In response, to complement the motion tracker results of Experiment 2, we conducted Experiment 3 using an RGB camera in conjunction with a posture estimation algorithm. The results indicate that the performance of the system with the RGB camera is comparable to that of the motion tracker, while maintaining a similar level of participant comfort. We believe this adequately addresses your concern by demonstrating the robustness of our approach (pp. 24-31, lines 424-557; pp. 35-36, lines 638-658).

[Rev#1C9] 8) I invite you to reconsider your conclusion (it is not well developed).

[Rev#1R9] Thank you for your suggestion to reconsider the conclusion. We have revised it to temper any strong claims regarding the effectiveness of the proposed system. The updated conclusion section now emphasizes the exploratory nature of the study and underscores the need for further research to validate the findings and investigate additional indicators of human behavior in response to AMRs. It also acknowledges the study’s limitations and provides a more balanced perspective on its contributions to future AMR development (pp. 37-38, lines 696-714).

-----

Reviewer #2:

[Rev#2C1] The authors presented an interesting work on AMR obstacle avoidance with humans. The study is timely as many AMRs are integrated into human environments and ensuring the safety of robots and humans is important. However, the study needs significant changes before publication.

[Rev#2R1] Thank you for your positive comments and constructive feedback. We have made substantial revisions to the manuscript in response to the reviewers’ comments and suggestions. Below, we provide detailed responses to the concerns raised and explain the changes made.

[Rev#2C2] 1. The Introduction section needs a better structure. Motivation and contributions are not mentioned clearly.

[Rev#2R2] Thank you for your feedback. We have revised the structure of the Introduction section to improve clarity (see also Rev#1R4). Additionally, we have clearly outlined our motivations in the subsection titled “Aims of This Study” and detailed our contributions in the Conclusion.

[Rev#2C3] 2. The literature survey should be added separately inside or after the Introduction section rather than citing it at different locations in the manuscript.

[Rev#2R3] Thank you for your suggestion. Accordingly, we have added a “Literature Review” section following the Introduction (see also Rev#1R5).

[Rev#2C4] 3. Why the data of one participant showed the opposite trend in Experiment 1? The issue was in data collection or data fitting?

[Rev#2R4] Thank you for your question regarding the outlier participant in Experiment 1. The opposite trend observed for this participant was attributed to differences in their behavior during data collection, rather than issues with data fitting. This participant exhibited a distinct trend compared to the others, potentially due to unique factors influencing their interaction with the AMR. We have acknowledged this outlier and carefully considered its impact on the overall analysis (pp. 12-13, lines 253-255).

[Rev#2C5] 4. Experiment 2 is confusing and needs a better structure for easier understanding. The concept of 0%, 50%, 100% and 150% is difficult to understand and the Fig. 5(b) isalso confusing.

[Rev#2R5] Thank you for your feedback regarding Experiment 2. To enhance clarity while maintaining consistency with the structure of Experiments 1 and 3, we have made minor adjustments rather than significant changes to the design of Experiment 2. Specifically, we have replaced the terminology for the concepts of 0%, 50%, 100%, and 150% with more intuitive terms, such as “beginning,” “early,” “same,” and “late.” Additionally, we have improved the interpretability of Fig. 5(b) by updating its x-axis labels to reflect the changes in terminology.

[Rev#2C6] 5. The Discussion Sections of each experiment and the General Discussion section can be merged into one.

[Rev#2R6] Thank you for your suggestion, which we have embraced by combining the individual discussions pertaining to each experiment with the general discussion into a single, unified section. Nevertheless, to enhance clarity, we have organized the content using subsections providing a detailed examination of each experiment while maintaining a cohesive overall discussion (pp. 31-37, lines 559-694).

Once again, we appreciate the opportunity to revise our manuscript based on the reviewers’ valuable input. We look forward to hearing from you regarding our submission and will be happy to respond should you have any further questions or comments.

Sincerely,

Tatsuto Yamauchi, Hideki Tamura, Tetsuto Minami, and Shigeki Nakauchi
