# Supplementary material for: Waist rotation angle as indicator of probable human collision-avoidance direction for autonomous mobile robots
Source: PLoS One. 2025 May 14;20(5):e0323632. doi: 10.1371/journal.pone.0323632 (PMC12077734; doi:10.1371/journal.pone.0323632)
Supplement: Attachment — Submitted filename: Yamauchi_mobility_response_R3_20250209.docx [file pone.0323632.s003.docx]

**Response Letter**

We thank the editor and reviewers for their time and effort in reviewing our manuscript. The changes we have implemented based on their insightful comments, suggestions, and feedback have significantly improved our work. We have made revisions to strengthen the manuscript. Additionally, we utilized a professional English language editing service to improve the language quality of the manuscript. Detailed responses to the reviewers’ comments are provided below.

Reviewer #1: This paper presents a series of experiments to study the interaction between AMR and human participants. The authors conducted three experiments to develop a method for collision avoidance that would be well perceived by human participants. In the first experiment human participants had to walk against AMR coming from different angles to study which behavior of human participants can hint for future actions. In particular, the authors intended to study whether it is possible to predict the direction in which people would move to avoid the AMR. Results showed that waist rotation occurs slightly before the actual steering maneuver and can therefore be used as a predictor for avoidance direction. Based on these results the authors implemented a collision avoidance algorithm in the robot and tested it in experiments. In the first experiment data from motion tracker on human were used to guide the robot. However, since this would not possible in reality the authors later also tested another method using information gained from a sensor on the robot. Results show that participants prefer an early steering maneuver from the robot and judge it more “comfortable.”

Thank you for summarizing our manuscript.

The paper is interesting and well presented. All details needed for reproducing the findings are provided and literature review is generally complete and satisfactory. The structure follows a logical structure in which exploratory experiments are presented first and implementation based on the results follow to show how the authors intend to make their solution usable in reality.

Thank you very much for your positive feedback.

However, I must admit I found the paper quite verbose. I understand that providing all details is important, but sometimes I found the amount of information overwhelming. For example (but it is not the only one), Fig. 6 contains all the value for the scale which is also reported in the text. I believe something like “from very fast to very slow” or “from very uncomfortable to very comfortable” would be enough.

Thank you for your specific feedback. We have simplified the y-axis label and caption in Figs. 6 and 8.

Also, much of the text in the discussion can be found in the presentation of the results. The fact that waist rotation can predict avoidance direction is repeated a large number of times in the manuscript and I am not sure whether it is really important to stress on something that is already clear when results are presented.

Thank you for your feedback. We have revised the discussion to eliminate redundancy with the results section.

Since this already the second review of this manuscript and I do not see the verbosity as an obstacle for publication (the paper is good, so for me can be published with minimal modification), I do not want to force the authors into an extensive round of review. Nonetheless, I believe that making the paper more “compact” can help them making it readable to a larger audience. For this reason I want to give a chance to the authors to review their paper, but I also want to make it clear it is not a condition for acceptance.

Thank you for your comments. As you suggested, we have eliminated redundant sentences. Additionally, we used an English proofreading service to ensure the coherence and flow of the text remain intact.

As a personal suggestion I would propose to only keep the aspects related to the experiment in the main text and move all minor technical details to an appendix. For example, average age of the participants, date of the experiment, ethical approval, OS and model of the robot, CV tool used, etc. can be moved to an appendix. I think they are not really relevant to understand the experiments and are only needed in case someone want to reproduce it (so they must be kept, but probably not in the main text).

Thank you for your suggestion. As you mentioned, we have reduced the main text in the Methods section and moved minor technical details to the Appendix to make the paper more concise. Specifically, parts of the *Participants* and *Apparatus* sections have been relocated. However, for Figs. 5 and 7, which illustrate the methodology, we retained the relevant descriptions in the main text to maintain consistency with the figure explanations, minimizing the information moved to the Appendix.

Some additional comments are given as follows:

1. Abstract: Readers cannot know that is your Experiment 2 and 3 so I suggest removing them. Also, since only experiments 2 and 3 are mentioned, one would wonder where it Experiment 1. Of course, that is clear in the manuscript, but abstract is read before the main text.

Thank you for your feedback. As you suggested, we have removed explicit references to Experiment 2 and Experiment 3 from the abstract to improve readability.

2. Fig. 5: I found if difficult to understand and generally not standalone. Maybe you can consider providing a number of snapshots, like in the old animation movies. Or use colors to show the change in time. For example, as robot and person move toward each other the color gets brighter (black, dark gray, light gray, etc.). b is also not that clear. The angle of the arrow changes a little, making it difficult to understand. What about using a top view where a person is moving the waist? In c I found “slower” and “more comfortable” redundant and creating more confusion.

Thank you for your suggestion. We have revised Fig. 5 as follows:

Fig. 5a: As you suggested, we illustrated the progression from the beginning to the end of a trial using snapshots from a video.

Fig. 5b: We clarified the movement of the participants’ waist by using a schematic top-down view, showing the changes in the waist angle (θ) for each condition (Beginning, Early, Same, Late).

Fig. 5c: We simplified the labels to improve readability.

3. Fig. 6: As already said, I found the reporting of the scale here redundant.

As mentioned above, we have revised the y-axis label and caption in Figs. 6 and 8.

4. Fig. 7: I believe you are using MATLAB. If so, why don’t you use unwrap to “join” the angles and avoid the jump from 0 to 360? You can have negative values and the graphs looks clearer. The jump you see looks like there is some data loss or something strange happened, while in reality is simply related to the definition of the angle.

Thank you for your suggestion. We applied the unwrap function in MATLAB to ensure a continuous representation of the angle without jumps. This adjustment makes Fig. 7 clearer and prevents any misinterpretation related to data loss or anomalies.

5. As already mentioned I do not want to force you into an additional work. I genuinely believe that the two papers below are related to your work, so maybe you can find them useful for this paper or in a follow-up study.

Jia, Xiaolu, et al. "Experimental study on the evading behavior of individual pedestrians when confronting with an obstacle in a corridor." Physica A: Statistical Mechanics and its Applications 531 (2019): 121735.

Murakami, Hisashi, et al. "Spontaneous behavioral coordination between avoiding pedestrians requires mutual anticipation rather than mutual gaze." Iscience 25.11 (2022).

Thank you for your suggestion. As you mentioned, the recommended papers are highly relevant to our manuscript, so we cited them in the main text (Murakami et al., 2022 in Literature review; Jia et al., 2019 in Discussion). We appreciate your insightful feedback.

Once again, we appreciate the opportunity to revise our manuscript based on the reviewers’ valuable input. We look forward to hearing from you regarding our submission and will be happy to respond should you have any further questions or comments.

Sincerely,

Tatsuto Yamauchi, Hideki Tamura, Tetsuto Minami, and Shigeki Nakauchi
