# Supplementary material for: Waist rotation angle as indicator of probable human collision-avoidance direction for autonomous mobile robots
Source: PLoS One. 2025 May 14;20(5):e0323632. doi: 10.1371/journal.pone.0323632 (PMC12077734; doi:10.1371/journal.pone.0323632)
Supplement: Attachment — Submitted filename: Yamauchi_mobility_response_R3_20250212.docx [file pone.0323632.s004.docx]

**Response Letter**

congratulation for the input otherwise I realized that the reviewer comments is not well answered kindly take you time and act on the below comments.

Thank you for reaching out to us. As outlined below, we have made the necessary revisions. We kindly ask for your review and confirmation.

Some additional comments are given as follows:

1. Abstract: Readers cannot know that is your Experiment 2 and 3 so I suggest removing them. Also, since only experiments 2 and 3 are mentioned, one would wonder where it Experiment 1. Of course, that is clear in the manuscript, but abstract is read before the main text.

Thank you for your feedback. As you suggested, we have removed explicit references to Experiment 2 and Experiment 3 from the abstract to improve readability.

2. Fig. 5: I found if difficult to understand and generally not standalone. Maybe you can consider providing a number of snapshots, like in the old animation movies. Or use colors to show the change in time. For example, as robot and person move toward each other the color gets brighter (black, dark gray, light gray, etc.). b is also not that clear. The angle of the arrow changes a little, making it difficult to understand. What about using a top view where a person is moving the waist? In c I found “slower” and “more comfortable” redundant and creating more confusion.

Thank you for your suggestion. We have revised Fig. 5 as follows:

Fig. 5a: As you suggested, we illustrated the progression from the beginning to the end of a trial using snapshots from a video.

Fig. 5b: We clarified the movement of the participants’ waist by using a schematic top-down view, showing the changes in the waist angle (θ) for each condition (Beginning, Early, Same, Late).

Fig. 5c: We simplified the labels to improve readability.

3. Fig. 6: As already said, I found the reporting of the scale here redundant.

We have revised the y-axis label and caption in Figs. 6 and 8.

4. Fig. 7: I believe you are using MATLAB. If so, why don’t you use unwrap to “join” the angles and avoid the jump from 0 to 360? You can have negative values and the graphs looks clearer. The jump you see looks like there is some data loss or something strange happened, while in reality is simply related to the definition of the angle.

Thank you for your suggestion. We applied the unwrap function in MATLAB to ensure a continuous representation of the angle without jumps. This adjustment makes Fig. 7 clearer and prevents any misinterpretation related to data loss or anomalies.

5. As already mentioned I do not want to force you into an additional work. I genuinely believe that the two papers below are related to your work, so maybe you can find them useful for this paper or in a follow-up study.

Jia, Xiaolu, et al. "Experimental study on the evading behavior of individual pedestrians when confronting with an obstacle in a corridor." Physica A: Statistical Mechanics and its Applications 531 (2019): 121735.

Murakami, Hisashi, et al. "Spontaneous behavioral coordination between avoiding pedestrians requires mutual anticipation rather than mutual gaze." Iscience 25.11 (2022).

Thank you for your suggestion. As you mentioned, the recommended papers are highly relevant to our manuscript, so we cited them in the main text (Murakami et al., 2022 in Literature review; Jia et al., 2019 in Discussion). We appreciate your insightful feedback.

Once again, we appreciate the opportunity to revise our manuscript based on the reviewers’ valuable input. We look forward to hearing from you regarding our submission and will be happy to respond should you have any further questions or comments.

Sincerely,

Tatsuto Yamauchi, Hideki Tamura, Tetsuto Minami, and Shigeki Nakauchi
