# Supplementary material for: Waist rotation angle as indicator of probable human collision-avoidance direction for autonomous mobile robots
Source: PLoS One. 2025 May 14;20(5):e0323632. doi: 10.1371/journal.pone.0323632 (PMC12077734; doi:10.1371/journal.pone.0323632)
Supplement: Attachment — Submitted filename: Yamauchi_mobility_response_R4_20250407.docx [file pone.0323632.s005.docx]

**Response Letter**

The term PSE (e.g., line 264 and elsewhere) is not appropriate as the term "subjective equality" is not well aligned with your analysis. I recommend revising it to something else. Given that your x-axis in Figure 3B indicats the robot's starting angle, you could use this term instead.

Thank you for pointing this out. We agree that the term “PSE” may not precisely reflect the nature of our analysis. In response to your suggestion, we have replaced it with either “starting angle” or “estimated starting angle,” depending on the context throughout the manuscript, including the caption of Figure 3B and the relevant sections in the main text.

Lines 316–317, consider using numbers ("reached 1 and 1.5 times the average range..").

Thank you for your suggestion. We have revised the sentence to use numeric expressions for clarity.

*“In the Early and Late conditions, the AMR initiated deflection when the participant’s waist rotation angle reached 0.5 and 1.5 times the average range of waist rotation angles, respectively.”*
